# Reverse engineering and identification in systems biology: strategies, perspectives and challenges

**DOI:** 10.1098/rsif.2013.0505

**Published:** 2014-02-06

**Authors:** Alejandro F. Villaverde, Julio R. Banga

**Affiliations:** BioProcess Engineering Group, IIM-CSIC, Spanish National Research Council, Vigo 36208, Spain

**Keywords:** systems biology, identification, inference, reverse engineering, dynamic modelling

## Abstract

The interplay of mathematical modelling with experiments is one of the central elements in systems biology. The aim of reverse engineering is to infer, analyse and understand, through this interplay, the functional and regulatory mechanisms of biological systems. Reverse engineering is not exclusive of systems biology and has been studied in different areas, such as inverse problem theory, machine learning, nonlinear physics, (bio)chemical kinetics, control theory and optimization, among others. However, it seems that many of these areas have been relatively closed to outsiders. In this contribution, we aim to compare and highlight the different perspectives and contributions from these fields, with emphasis on two key questions: (i) why are reverse engineering problems so hard to solve, and (ii) what methods are available for the particular problems arising from systems biology?

## 1. Introduction

In the late 1960s, Mesarović [1, p. 83] stated something that is still relevant today: ‘the real advance in the application of systems theory to biology will come about only when the biologists start asking questions which are based on the system-theoretic concepts rather than using these concepts to represent in still another way the phenomena which are already explained in terms of biophysical or biochemical principles’.

Four decades later, Csete & Doyle [2], considering the reverse engineering of biological complexity, argued that, although biological entities and engineered advanced technologies have very different physical implementations, they are quite similar in their systems-level organization. Furthermore, they also noted that the level of complexity in engineering design was approaching that of living systems. When viewed as networks, biological systems share some important structural features with engineered systems, such as modularity, robustness and use of recurring circuit elements [3]. Frequently, important aspects of the functionality of a network can be derived solely from its structure [4]. It seems therefore natural that systems engineering and related disciplines can play a major role in modern systems biology [5–9].

Today, a decade after the reverse engineering paper of Csete & Doyle, recent research [10] clearly shows the feasibility of comprehensive large-scale whole-cell computational modelling. This class of models includes the necessary detail to provide mechanistic explanations and allows for the investigation of how changes at the molecular level influence behaviour at the cellular level [11]. Multi-scale modelling, which considers the interactions between metabolism, signalling and gene regulation at different scales both in time and space, is key to the study of complex behaviour and opens opportunities to facilitate biological discovery [12,13]. The interplay between experiments and computational modelling has led to models with improved predictive capabilities [14]. In the case of evolutionary and developmental biology, reverse engineering of gene regulatory networks (GRNs) and numerical (*in silico*) evolutionary simulations have been used [15,16] to explain observed phenomena and, more importantly, to suggest new hypotheses and future experimental work. Finally, model-based approaches are already in place for the next step, namely, synthetic biology [17].

Most reverse engineering studies of biological systems have considered microbial cells. In this context, a wide range of modelling approaches have been adopted, which can be classified according to different taxonomies. Stelling [18] distinguished between three large groups: interaction-based (no dynamics, no parameters [19,20]), constraint-based (no dynamics, only stoichiometry parameters [21,22]) and mechanism-based models (dynamic, with both stoichiometry and kinetic parameters). Other classifications can be found in more recent literature, such as those based on modelling formalisms [23,24], which include Boolean networks, Bayesian networks (BNs), Petri nets, process algebras, constraint-based models, differential equations, rule-based models, interacting state machines, cellular automata and agent-based models.

Regardless of the type of representation chosen, the importance of taking into account the system dynamics has to be acknowledged [25,26]. It has been stated that the central dogma of systems biology is that the functioning of cells is a consequence of system dynamics [5]. In particular, regulation—usually achieved by feedback—plays a key role in biological processes [27]. Hence, the study of the rich behaviour exhibited by biological systems requires the use of engineering tools, namely from the systems and control areas [7]. Furthermore, it has been argued that even more interesting than the application of systems engineering ideas to biological problems is the inspiration that these problems provide in the development of new theories [8]. Systems engineering aims to design systems, while biology aims to understand (reverse engineering) them; it is natural then that these two communities have traditionally specialized in solving different problems. However, the interplay between both disciplines can be mutually beneficial [6]; in this sense, systems biology can be seen ‘not as the application of engineering principles to biology but as a merger of systems and control theory with molecular and cell biology’ [5].

This work reviews different perspectives for the reverse engineering problem in biological systems. The first step in the identification of a dynamic model is to establish its components and connectivity, a task for which either prior knowledge or data-driven statistical methods is required [28]. We begin by discussing these methods in §2, where we address the reduced problem of recovering interaction structures. We classify the methods proposed for this task in three main strategies: correlation-based, information-theoretic and Bayesian. Then in §3, we discuss the different perspectives for the reverse engineering of complete dynamic models,^1^ grouping them in eight areas: inverse problems, optimization, systems and control theory, chemical reaction network theory, Bayesian statistics, physics, information theory and machine learning. We finish this review with some conclusions about the convergence of these perspectives in §4.

### 2. Interaction networks: three main strategies

We address now the question of reverse engineering systems modelled as interaction networks. This problem can be formulated as follows: given a list of nodes (variables), infer the connections (dependencies) among them using the information contained in the available datasets. The goal is the determination of the existing interactions, not the detailed characterization of these interactions. Thus, the recovered models do not include differential equations, and there is no need to estimate parameter values such as kinetic constants. This problem can be considered as a reduced version of the general reverse engineering problem, which will be considered in the following sections. However, this does not mean that it is easy to solve; on the contrary, it is still a very active area of research. The key task is to estimate the strength of the dependence among variables using the available data.

Most of the methods used to infer interactions are ultimately related to statistics. In this context, it is worth mentioning that there are several schools of thought in statistics: Bayesian, frequentist, information-theoretic and likelihood (the latter being a common element in all of them). However, roughly speaking, the Bayesian and frequentist approaches are usually considered the main paradigms [33,34].

The history of statistics reveals that the Bayesian approach was initially developed in the eighteenth century. Bayes himself only considered a special case of the theorem that receives his name, which was actually rediscovered independently and further developed in its modern form by Laplace years later. At that time, the theory received the name of inverse probability. Frequentist statistics was developed during the first decades of the twentieth century by Pearson, Neyman and Fisher, among others. The frequentist theory rapidly displaced the inverse probability (Bayesian) approach and became the dominant school in statistics. Bayesian ideas barely survived, mostly outside statistics departments (a detailed history is given in [35]). The use of Bayes prior information was regarded by many frequentists as the introduction of subjectivity, and therefore, a biased approach, something not acceptable in the scientific method. Although refinements, e.g. empirical Bayes methods [36] (prior distribution based on existing data, not assumptions), tried to surmount this, Bayesian approaches still had another major problem: the computations needed were extremely demanding.

In the 1980s, the application of Markov chain Monte Carlo (MCMC) methods [37,38] changed everything. MCMC and related techniques [39,40] made feasible many of the complex computations necessary in Bayesian methods and the theory resurfaced and started to be applied in many areas [41], including bioinformatics and computational systems biology [42–44].

**Figure 1.**
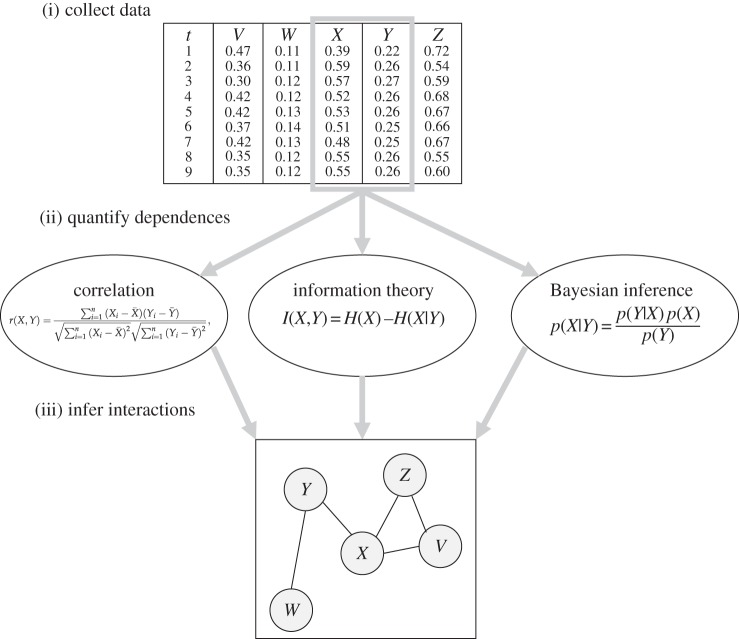
Approaches for inferring interaction networks. Schematic of the process of inferring a network structure from data, showing three approaches for measuring dependence among variables: correlation-based, information theoretic and Bayesian.

Depending on the statistic used to measure the interaction strength, the most common reverse engineering approaches can be classified into three classes: correlation, mutual information and Bayesian (see figure 1). Their main characteristics are discussed in the following subsections; more detailed surveys can be found in [45–49]. With a more specific focus, Bayesian methods were covered in [50,51] and information-theoretic approaches in [52] (figure 1).

### 2.1. A classical tool: correlation

The correlation coefficient *r*, commonly referred to as the Pearson correlation coefficient, quantifies the dependence between two random variables *X* and *Y* as2.1
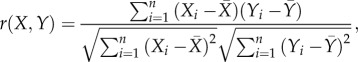
where *X_i_*, *Y_i_* are the *n* data points and 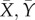 are their averages. If both variables are linearly independent, *r*(*X*,*Y*) = 0; in the opposite situation, where one variable is completely determined by the other, *r*(*X*,*Y*) = ±1.

Correlation-based methods can be used for unsupervised learning from data and have been widely used to discover biological relationships. While most applications have been developed for genetic networks [53,54], there are also examples in reverse engineering metabolic networks. One such method is correlation metric construction [55], which takes into account time lags among species and was successfully tested on the glycolytic pathway [56]. A more sophisticated measure of association between variables is the distance correlation method [57,58], which has theoretical advantages over Pearson's coefficient and has been recently used in biological applications [59,60].

### 2.2. Perspective from information theory

While the Pearson coefficient is appropriate for measuring linear correlations, its accuracy decreases for strongly nonlinear interactions. A more general measure is mutual information, a fundamental concept of information theory defined by Shannon [61]. It is based on the concept of entropy, which is the uncertainty of a single random variable: let *X* be a discrete random vector with alphabet χ and probability mass function *p*(*x*). The entropy is2.2
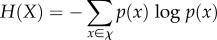
and the conditional entropy *H*(*Y*|*X*) is the entropy of a variable *Y* conditional on the knowledge of another variable *X*2.3
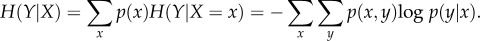
The mutual information *I* of two variables measures the amount of information that one variable contains about another; equivalently, it is the reduction in the uncertainty of one variable owing to the knowledge of another. It can be defined in terms of entropies as [62]2.4
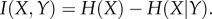


As mutual information is a general measure of dependencies between variables, it may be used for inferring interaction networks: if two components have strong interactions, their mutual information will be large; if they are not related, it will be theoretically zero. Mutual information has been applied for reverse engineering biological networks since the 1990s. In early applications [63–67], genetic interactions were hypothesized from high values of pairwise mutual information between genes. The success of this approach encouraged further research and increasingly sophisticated techniques were developed during the following decade. One of the most popular methods for GRN inference is ARACNE [68], which exploits the data processing inequality (DPI, [62]) to discard indirect interactions. The DPI states that if *X* → *Y* → *Z* is a Markov chain, then *I*(*X*,*Y*) ≥ *I*(*X*,*Z*). ARACNE examines the gene triplets (*X*,*Y*,*Z*) that have a significant value of mutual information and removes the edge with the smallest value, thus reducing the number of false positives. A time-delay version of ARACNE, which is especially suited for time-course data, is also available [69].

In reverse engineering applications, the probability mass functions *p*(*x*), *p*(*y*) are generally unknown; however, they can be estimated from experimental data using several methods. The simplest one is to partition the data into bins of a fixed width, and approximate the probabilities by the frequencies of occurrence. This naive solution has the drawback that the mutual information is systematically overestimated [70]. To avoid this problem, one can either make the bin-size dependent on the density of data points (adaptive partitioning, [71]), or use kernel density estimation [72]. The influence of the choice of estimators on the network inference problem has been studied in [73].

Information-theoretic methods have a rigorous theoretical foundation on concepts that allow for an intuitive interpretation. This facilitates the development of new methods that are aimed at specific purposes. An example is the distinction between direct and indirect interactions, which has motivated the design of methods, such as minimum redundancy networks [74], three-way mutual information [75], entropy metric construction and entropy reduction technique [76], among others. Another example is the modification of the calculation of mutual information by taking into account the background distribution for all possible interactions, as done by the context likelihood of relatedness technique (CLR) [77]. The combination of CLR with another method, the Inferelator [78], became one of the top performers at the DREAM4 100-gene *in silico* network inference challenge [79]. In yet another example, a recently presented statistic called maximal information coefficient [80] aims to enforce equitability, a property that consists of assigning similar values to equally noisy relationships, independently of the type of association.

Cantone *et al.* [81] argued in 2009 that information-theoretic methods were not appropriate for reconstruction of small networks, because they could not infer the direction of regulations. However, in the years following that statement some progress was made, and some information-theoretic methods capable of recovering directions are already available [69,82].

### 2.3. Incorporating prior knowledge: the Bayesian inference perspective

Prior knowledge can be incorporated into the inference procedure using a Bayesian framework. The Bayes rule for two variables *X* and *Y* is2.5
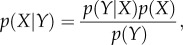
where *p*(*Y*) and *p*(*X*|*Y*) are called prior and posterior probabilities, respectively. In a typical scenario, *X* may be the value of a parameter and *Y* the available data. The Bayes rule allows the belief in a prediction to be updated given new observations. In practice, complications may arise owing to the fact that typically neither *p*(*Y*) nor *p*(*Y*|*X*) is known. Estimation of these quantities usually involves computationally costly calculations, which do not scale up well for large networks. It may be necessary to decompose the full problem according to the underlying conditional independence structure of the model [42]; graphical models appear in this context. Probabilistic graphical models represent joint probability distributions as a product of local distributions that involve only a few variables [83]. BNs are probabilistic graphical models in which the variables are discrete; their graphical representation is given by a directed acyclic graph (DAG).

BNs can be automatically inferred from data, a problem known as Bayesian inference. Reverse engineering a BN consists of finding the DAG that ‘best’ describes the data. The goodness of fit to the data is given by a score calculated from the Bayes rule. It should be noted that the search for the best BN is an NP-hard problem [84], and therefore heuristic methods are used for solving it. Additionally, it is possible to look for approximations that help to decrease the computational complexity: approximate Bayesian computation (ABC) methods estimate posterior distributions without explicitly calculating likelihoods, using instead simulation-based procedures [85,86]. A Bayesian method for constructing a probabilistic network from a database was first presented in [87].

Genetic networks can be represented as probabilistic graphical models, by associating each gene with a random variable. The expression level of the gene gives the value of this random variable. Bayesian approaches were first used for reverse engineering genetic networks from expression data in [88]. An important limitation of BNs is that they are acyclic, while in reality most biological networks contain loops. An extension of BNs called dynamic Bayesian networks (DBNs) can be used to overcome this issue. Unlike BNs, DBNs can include cycles and may be constructed when time-course data are available [89–92].

## 3. Dynamic models: perspectives from different areas

Here, we focus on dynamic (kinetic) models of biological systems. These models typically consist of systems of differential equations. From the identification point of view, one can distinguish among three main problem classes (in decreasing order of generality):

(1) Full network inference (reverse engineering or reconstruction): given (high-throughput) dynamic data (i.e. time-series of measured concentrations and other properties), one seeks to find the full network (kinetic model structure and kinetic parameters) that fits (explains) the data.
(2) Network selection (network refinement, retrofitting): given dynamic data and an existing dynamic model with possible structural modifications (or a set of alternative kinetic model structures), the objective is to find the structural modifications and the kinetic parameters that fit the data.
(3) Kinetic parameter estimation (model calibration, parametric identification): given dynamic data and a fixed kinetic model structure, the objective is to find the kinetic parameters that fit the data.

Problem (1) above is the most general, while problem (2) is somewhere in the middle between the general inference problem and the more focused parameter estimation problem. Although fitting existing data is usually the first objective sought, one should also perform cross-validation studies with a different set of existing data. Ultimately, one should also seek to use the inferred model to allow for high-quality predictions under different conditions. Problem (1) has been usually solved using a bilevel approach, first determining the interaction network (as discussed in §2), and then identifying the kinetic details.

It has been widely recognized that all of the above problems are hard. Many approaches have been proposed for solving them, using different theoretical foundations. Several authors have carried out comparisons among methods using simulated or experimental data; early examples can be found in [46,93]. It is particularly interesting to explore the conclusions of organizers of the DREAM (Dialogue for Reverse Engineering Assessment and Methods) challenge, which is probably the best current source of comparisons of different methods. The DREAM challenges take place annually and they seek to promote the interactions between theoretical and experimental methods in the area of cellular network inference and model building. In Prill *et al.* [94], the organizers state: ‘The vast majority of the teams’ predictions were statistically equivalent to random guesses. Moreover, even for particular problem instances like gene regulation network inference, there was no one-size-fits-all algorithm’. In other words, reliable network inference remains an unsolved problem. The organizers identify two major hurdles to be surmounted: lack of data and deficiencies in the inference algorithms. We agree with this diagnostic but, as we show below, we also think that there are other hurdles that are as important and that have been mostly ignored until recently.

**Figure 2.**
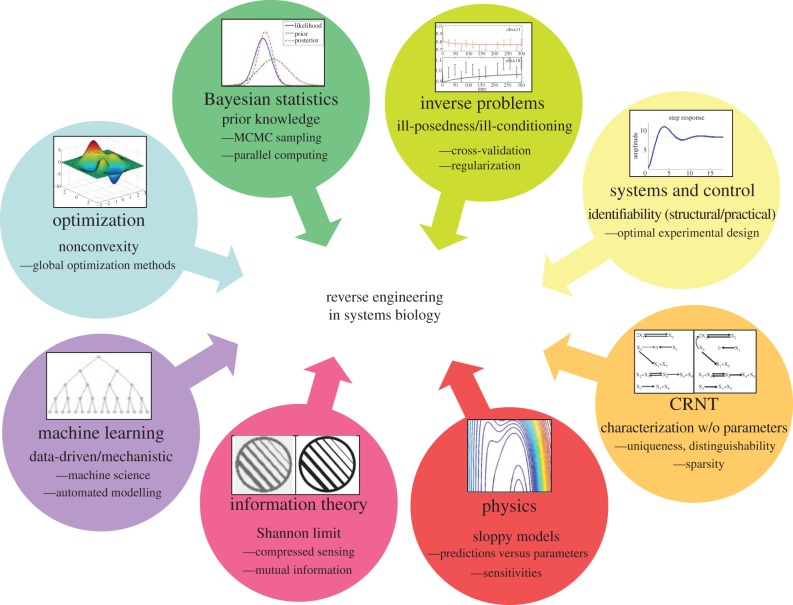
Perspectives on reverse engineering. An overview of the different perspectives that converge in the area of systems biology, showing some of their key concepts and tools. (Online version in colour.)

The above problems are obviously not exclusive of systems biology and have been (and continue to be) studied in different areas, such as statistics, machine learning, artificial intelligence, nonlinear physics, (bio)chemical kinetics, systems and control theory, optimization (local and global), inverse problems theory, etc. (figure 2). This is a rather *ad hoc* list, because there is significant overlap between these disciplines, and some people might claim that some are simply subareas of others. However, our intention here is not to come up with a consensus classification but rather to highlight that these different areas (or, maybe better, communities) have looked in depth at the reverse engineering problem during the last decades, arriving at several powerful principles. However, despite the interdisciplinary nature of systems biology, these different perspectives have apparently not exchanged notes to the degree that one might expect for such a general problem.

In the following, we intend to give the reader the principal components of these different perspectives. With the aim of facilitating the readability of the associated literature, the presentation of the different perspectives is ordered according to the timeline of their key developments. In particular, we want to consider the different answers to two main questions:

— Why are the problems (1–3) above so challenging?
— Which methods are available to solve them?

### 3.1. Perspective from inverse problems

Inverse problem theory [95,96] is a discipline that aims to find the best model to explain (at least in an approximate way) a certain set of observed data. The name comes from the fact that it is the reverse of the direct (or forward) problem, i.e. given a model and its parameters, generate predictions by solving the model. Hadamard [97] was already aware of the difficulties associated with such an exercise, and defined well-posed problems as those with the following properties:

— existence: a solution exists;
— uniqueness: the solution is unique; and
— stability: the solution's behaviour hardly changes when there is a slight change in the initial condition or parameters (the solution depends continuously on the data).

Inverse problems are often ill-posed in the sense of Hadamard. Furthermore, many problems are well-posed but ill-conditioned, meaning that the solution of the inverse problem is very sensitive to errors and noise in the data. In these situations, solving the original problem can result in overfitting, i.e. the fitted model will describe the noise instead of the underlying relationship. An overfitted model might be able to describe the data well but will have poor predictive value. This situation can be avoided by using cross-validation and/or regularization methods.

Cross-validation [98,99] tries to estimate the performance of a predictive model in practice. In its simplest form, the available data are partitioned into two subsets, using the first to solve the inverse problem, and then evaluating its predictive performance with the second subset.

Regularization tries to reduce the ill-conditioning by introducing additional information via a penalty function in the cost term to be minimized. For linear systems, Tikhonov regularization [100] is the most popular approach. For nonlinear dynamical systems, it remains an open question, although successful applications of Tikhonov-inspired schemes have been reported. Engl *et al.* [101] review these topics in the context of systems biology and present results supporting the use of sparsity-enforcing regularization. We will revisit the sparsity-enforcing concept and its consequences below.

### 3.2. Perspective from optimization

Identification problems are usually formulated using an optimization framework, seeking to minimize a cost function which is a metric of the distance between the predicted values and the real data. Convex optimization [102] problems have nice properties: the minimum is unique and algorithms for solving them scale up well with problem size. However, the identification of nonlinear dynamic models results in non-convex problems, which exhibit a wide range of possible pitfalls and difficulties [103] when one attempts to solve them with standard local optimization methods: convergence to local solutions, badly scaled and non-differentiable model functions, flat objective functions in the vicinity of solutions, etc. Therefore, the use of popular local methods, such as Levenberg–Marquardt or Gauss–Newton, will result in different solutions depending on the guess for the starting point in the parameter space.

It is sometimes argued that these difficulties can be avoided by using a local method in a multi-start fashion (i.e. repeated solutions of the problem starting with different guesses of the parameters). However, this folklore approach [104] is neither robust (it fails with even small problems) nor efficient (the same local optima are found repeatedly since many of the initial guesses are inside the same basins of attraction of local minima).

As a consequence, there is a need for proper non-convex (global) optimization methods [105,106]. Deterministic approaches for global optimization in dynamic systems [107,108] can guarantee the global optimality of the solution, but the associated computational effort increases very rapidly with problem size. This is a consequence of the NP-hard nature of these problems. In fact, global optimization problems are undecidable in unbounded domains [109], and NP-hard on bounded domains [110]. Therefore, based on the current status of the NP issue [111], approximate methods (such as stochastic algorithms and metaheuristics) are a more attractive alternative for problems of realistic size [112–114]. The price to pay is the lack of guarantees regarding the global optimality of the solution found. However, as the objective function to be minimized has a lower bound, which can be estimated from *a priori* considerations, obtaining a value close to that bound gives us enough indirect confidence of the near-global nature of a solution. These methods have been successfully applied to different benchmark problems with excellent results [115]. Moreover, they can be parallelized, so their application to large-scale kinetic models is feasible [116]. Further computational efficiency can be gained by following divide and conquer strategies [117].

A common question in this context is to identify the best performing method to solve a particular global optimization problem. Wolpert & Macready [118] caused quite a stir with the publication of the NFL (no free lunch) theorem. Basically, the theorem shows that if method A outperforms method B in solving a certain set of problems, then B will outperform A in a different set. Thus, considering the space of all possible optimization problems, all methods are equally efficient (so there is no free lunch in optimization). A number of misconceptions from this theorem were derived by others, including (i) the claim that there is no point in comparing metaheuristics for global optimization, as there can be no winner owing to NFL and (ii) the whole enterprise of designing global optimization methods is pointless owing to the NFL nature of optimization. What is fundamentally wrong in these claims is that the NFL theorem considers ALL possible problems in optimization, which is certainly not the case in practical applications such as parameter estimation. Furthermore, the theorem considers methods without resampling, an assumption not met by most modern metaheuristics. Finally, many modern metaheuristics exploit the problem structure to increase efficiency. For example, scatter search has proved to be a very efficient method when the local search phase is performed by a specialized local method [114,116]. Again, in these conditions, the NFL theorem does not apply.

The above does not mean in any way that global optimization problems cannot be extremely hard. It is quite easy to build a needle-in-a-haystack type of problem, which will be pathologically difficult for any algorithm, because it has no structure, and therefore requires full exploration of the search space (or a lot of luck). For this type of problem, it becomes obvious that on average, no method will perform better than pure random search, and therefore we might be tempted to assume that the NFL theorem is right after all. Fortunately, needle-in-a-haystack problems do not appear in practice, and if they do, they will very likely be the consequence of extremely poor modelling.

In summary, the NFL theorem can be regarded as one of those impossibility theorems which, although true for the general assumptions considered, do not really have major implications in a real practice framework, and therefore it offers a pessimistic view which is the consequence of its universality (‘all possible problems’). This is similar to Godel's incompleteness theorems, which have not stopped advances in mathematics [119]. As we will see below with yet another impossibility theorem, the fact that our practical problems have a structure that can be exploited allows us to escape from such a pessimistic trap.

### 3.3. Perspective from systems and control theory

System identification theory [120,121] was developed and applied in the control engineering field with the purpose of building dynamic models of systems from measured data. This theory is well developed for linear systems, but remains as a very active research area for the nonlinear dynamic case [122].

Although the systems and control area has been primarily focused on engineered systems (mechanical, electrical and chemical), it also has a long record of applications in biology. For example, back in 1978 Bekey & Beneken [123] published a review paper on the identification of biological systems. In fact, we could also consider the pioneer contributions of Wiener [124] and Ludwig von Bertalanffy [125] as seminal examples of the interactions between biology and systems and control theory. It has been increasingly noted that these interactions can be instrumental in solving relevant problems in areas, such as medicine and biotechnology [126].

A key concept in system identification is the property of identifiability: roughly speaking, a system is identifiable if the parameters can be uniquely determined from the given input/output information (data). One can distinguish between structural [127] and practical identifiability [128]. In the structural case, identifiability is a property of the model structure (its dynamics), and the observation and stimuli (control inputs) functions (perfect measurements are assumed). In the case of practical identifiability, the property is related to the experimental data available (and their information content). Despite its importance, most modelling studies in systems biology have overlooked identifiability. Fortunately, recent literature is correcting this (e.g. [129–140]). Despite the frequent problems of lack of full identifiability, models can still be useful to predict variables of interest [141,142]. To address the issues of sparse and noisy data, Lillacci & Khammash [143] propose a combination of an extended Kalman filter (a recursive estimator well known in control engineering) with *a posteriori* identifiability tests and moment-matching optimization. The resulting approach may be used for obtaining more accurate estimates of the parameters as well as for model selection.

A closely related topic is that of optimal experimental design (OED), i.e. how we should design experiments that would result in the maximum amount of information so as to identify a model with the best possible statistical properties (which are user defined and can be related to precision, decorrelation, etc.). The advantages for efficient planning of biological experiments are obvious and have been demonstrated in real practice. For example, Bandara *et al.* [144] showed how two cycles of optimization and experimentation were enough to increase parameter identifiability very significantly. The topic of optimal design of dynamic experiments in biological systems is receiving increased attention [144–152]. Balsa *et al.* [145] presented computational procedures for OED, which was formulated as a dynamic optimization problem and solved using control vector parametrization. He *et al.* [148] compared two robust design strategies, maximin (worst-case) and Bayesian, finding a trade-off between them: while the Bayesian design led to less conservative results than the maximin, it also had a higher computational cost.

Improving the quality of parameter estimates is not the only purpose of OED; it can also be used for inferring the network topology. Tegnér *et al.* [153] proposed a reconstruction scheme where genes in the network were iteratively perturbed, selecting at each iteration the perturbation that maximized the amount of information of the experiment.

Another common application of OED is discrimination among competing models [147]. With this aim, Apgar *et al.* [129] proposed a control-based formulation, where the stimulus is designed for each candidate model so that its outputs follow a target trajectory; the quality of a model is then judged by its tracking performance. In [149], three different approaches were considered, each of which optimized initial conditions, input profiles or parameter values corresponding to structural changes in the system. Other methods have exploited sigma-point approaches [151] or Kullback–Leibler optimality [150,152].

OED with dynamic stimuli is therefore a powerful strategy to maximize the informative value of experiments while minimizing their number and associated cost. Ingolia & Weissman [154] highlight the importance of choosing the way to perturb biological systems, because it determines what characteristics of those systems can be observed and analysed, as illustrated in [155,156]. In summary, there is a need for technologies that permit a wide range of perturbations and for OED methods which can make the most out of them.

A topic that deserves special attention is the analysis of kinetic models under uncertainty. Kaltenbach *et al.* [157] offer an interesting study focused on epistemic uncertainty (lack of knowledge about the cellular networks) owing to practical limitations. These authors support the idea that the structure of these networks is more important than the fine tuning of their rate laws or parameters. As a result, methods that are based on structural properties are able to extract useful information even from partially observed and noisy systems. Kaltenbach *et al.* [157] also offer an excellent overview of methods from different areas, noting the ‘cultural’ differences that need to be addressed in systems biology. Vanlier *et al.* [140] provide an introduction to various methods for uncertainty analysis (focusing on parametric uncertainty). In addition to giving an overview of current methods (including frequentist and Bayesian approaches), these authors highlight how the applicability of each type of method is linked to the properties of the system considered and the assumptions made by the modeller. This type of study is of great interest as it provides system biologists with a balanced view of the requirements and results that are expected in each method. Ensemble modelling is a particularly interesting type of Monte Carlo methodology that has been used to account for uncertainty in many areas, from weather forecasting to machine learning. Applications in systems biology have already appeared [158,159]. Another related successful approach for robust inference is the wisdom of crowds [160].

Finally, advances in the identification of biological systems ultimately lead to their control [8], and here the possibilities are enormous, especially in synthetic biology [161–167].

### 3.4. Perspective from chemical reaction network theory

The fundamentals of chemical reaction network theory (CRNT) were established back in the 1970s by Horn, Jackson and Feinberg [168–170]. The theory remained rather dormant until authors like Bailey [171] highlighted its potential for the analysis of biological networks. The basic idea is that, using CRNT, we can characterize kinetic models (multi-stability, oscillations, etc.) without knowing the precise values of the kinetic parameters. During the last decade, research based on CRNT has gained momentum [172–178] leading to major contributions [179].

Regarding the identification of biological systems, CRNT offers several results of considerable importance. Craciun & Pantea [180] make use of CRNT to show that, given a (mass action) reaction network and its dynamic equations (ODEs), it might be impossible to identify its rate constants uniquely (even with perfect measurements of all species). Furthermore, they also show that, given the dynamics, it might be impossible to identify the reaction network uniquely.

Szederkenyi *et al.* [181] make use of CRNT principles to explore inherent limitations in the inference of biological networks. Their results show that, in addition to the obstacles identified by Prill *et al.* [94] (lack of data and deficiencies in the inference algorithms), we must be also aware of fundamental problems related to the uniqueness and distinguishability of these networks (even for the utopian case of fully observed networks with no noise). More importantly, uniqueness and distinguishability of models can be guaranteed by carefully adding extra constraints and/or prior knowledge. A topic that deserves further investigation is the effect of imposing a sparse network topology. Data from cellular networks suggest such a sparse topology, so it is a common prior enforced in many inference methods [182,183]. However, Szederkenyi *et al.* [181] show that the sparsity assumption alone is not enough to ensure uniqueness. Moreover, in the case of linear dynamic genetic network models, too sparse structures can be harmful.

### 3.5. Perspective from Bayesian statistics

As previously mentioned, the origin of the Bayesian approach goes back to the eighteenth century, and the statistical methods used during the nineteenth century were mostly Bayesian as well. However, during the twentieth century the frequentist paradigm clearly dominated statistics [184]. Frequentism was the default approach used for estimation and inference of kinetic (dynamic) models, where most studies (cited in the previous subsections) considered maximum-likelihood and related metrics as the cost functions to optimize. However, fuelled by important developments in MCMC methods in the 1990s, the beginning of the twenty-first century witnessed a Bayes revival, and studies on Bayesian methods for dynamic models started to appear as a result of theoretical and computational advances and the greater availability of more powerful computers. In parallel, systems biology was taking off with the new century, requiring methods that were able to handle the biological complexity. Bayesian methods, which are especially useful to extract information from uncertain and noisy data (the most common scenario in bioinformatics and computational systems biology), started to receive greater attention [42,44,185]. Bayesian estimation in stochastic kinetic models was considered in several seminal works regarding diffusion models [186,187]. Similarly, in the case of deterministic kinetic models, the last decade has seen a rapidly growing Bayesian literature. Pioneering work using Monte Carlo methods were presented by Battogtokh *et al.* [188] and Brown & Sethna [189]. Sanguinetti *et al.* [190], considering a discrete time state space model, presented a Bayesian method for genome-wide quantitative reconstruction of transcriptional regulation. Girolami [191] illustrated the use of the Bayesian framework to systematically characterize uncertainty in models based on ordinary differential equations. Vyshemirsky & Girolami [192] compared four methods for estimating marginal likelihoods, investigating how they affect the Bayes factor estimates, which are used for kinetic model ranking and selection.

When the formulation of a likelihood function is difficult or impossible, ABC-like approaches can be adopted [85]. ABC schemes replace the evaluation of the likelihood function with a measure of the distance between the observed and simulated data. Briefly, ABC algorithms sample a parameter vector from the distribution and use it for generating a simulated dataset. Then, they calculate the distance between this dataset and the experimental data, and if it is below a certain threshold they accept the candidate parameter vector. The weakness of this approach, at least in its simplest form, is that it can have a low acceptance rate when the prior and posterior are very different. To overcome this problem, Marjoram *et al.* [193] presented a MCMC algorithm (ABC MCMC) that accepts observations more frequently and does not require the computation of likelihoods. The price to pay is the generation of dependent outcomes, and the risk of getting stuck in regions of low probability of the state space for long periods of time. An alternative is to use sequential Monte Carlo (SMC) techniques, which use an ensemble of particles to represent the posterior density, with each sample having a weight that represents its probability of being sampled. SMC particles are uncorrelated, and the approach avoids being stuck in low probability regions. Sisson *et al.* [194] proposed a likelihood-free ABC sampler based on SMC simulation (ABC SMC) and a related formulation was proposed by Toni *et al.* [195,196], who applied it for parameter estimation and model selection in several biological systems.

ABC schemes can also be used to improve computational efficiency, which is an important issue in Bayesian approaches. Using the full probability distribution of parameters instead of single estimates of parameter values entails calculating the likelihood across the whole parameter space, a step that can be very costly.

The availability of these theoretical and computational advances has led to their successful application in combination with biological experimentation. For example, Xu *et al.* [197] considered the ERK cell signalling pathway and found unexpected new results of biological significance, demonstrating the capability of Bayesian approaches to infer pathway topologies in practical applications, even when measurements are noisy and limited. In another recent application, Eydgahi *et al.* [198] used Bayes factor analysis to discriminate between two alternative kinetic models of apoptosis. It is interesting to note that the approach allowed these authors to assign a much greater plausibility to one of the models even though both presented equally good fits to data. Moreover, it is also remarkable that, despite non-identifiability of the models, the Bayesian approach resulted in predictions with small confidence intervals. Regarding experimental design, Liepe *et al.* [199] illustrated the combination of Bayesian inference with information theory to design experiments with maximum information content and applied it to three different problems.

Recently, Raue *et al.* [200] presented an interesting study combining the frequentist and the Bayesian approaches. These authors note that for kinetic models with lack of identifiability (structural and/or practical), the Markov chain in MCMC-based Bayesian methods cannot ensure convergence and will result in inaccurate results. To surmount this, they suggest a two-step procedure. In the first step, a frequentist profile-likelihood approach is used in iterative combination with experimental design until the identifiability problems are solved. Then, in the second step, the MCMC approach can be used reliably.

Another important question concerns the scalability of Bayesian approaches, i.e. can they handle large-scale kinetic models? In a recent contribution, Hug *et al.* [201] discuss the conceptual and computational issues of Bayesian estimation in high-dimensional parameter spaces and present a multi-chain sampling method to address them. The feasibility and efficiency of the method is illustrated with a signal transduction model with more than 100 parameters. The study is a significant proof of principle and also a good example of the care that must be taken regarding the verification of results.

The existing literature indicates the importance of adequate selection of priors. Gaussian processes (GPs) can be used for specifying a prior directly over the function space, which is often simpler than over the parameter space. A GP [202] is a stochastic process for which any set of variables have a joint multi-variate Gaussian distribution. Gaussian processes are generalizations of Gaussian probability distributions: they describe the properties of functions rather than of scalars or vectors. They have also been applied in the development of efficient and reliable sampling schemes. Here, Calderhead *et al.* [203] illustrated how GPs can be used to greatly accelerate Bayesian inference in nonlinear dynamic models. Other notable recent advances in sampling methods have been presented by Girolami & Calderhead [204,205] and Schmidl *et al.* [206].

### 3.6. Perspective from physics

Physics has made numerous and highly relevant contributions to inference and mathematical modelling in general. In fact, the origins of many of the ideas classified in the sections above can be traced back to developments in physics. Therefore, our intention here is not to present any type of overview of such vast history.

Rather, we will focus on recent research that has spurred a broad discussion about whether there exist fundamental limitations regarding dynamic modelling of biological systems. Gutenkunst *et al.* [207] discuss the concept of sloppy models (introduced by Brown & Sethna [189]), i.e. multi-parametric models whose behaviour (and predictions) depends only on a few combinations of parameters, with many other sloppy parameter directions which are basically unimportant. These authors tested a collection of 17 systems biology models and concluded that (i) sloppiness is universal in systems biology models and (ii) sloppy parameter sensitivities help to explain the difficulty of extracting precise parameter estimates from collective fits, even from comprehensive data. The previous study by Brown & Sethna [189] presents a sound theoretical analysis based on statistical thermodynamics and Bayesian inference.

This work has received great attention from the systems biology community. Here, we would like to highlight some open questions to be addressed, and comment on possible misconceptions surrounding it. Some of our remarks below can also be found in the correspondence by Apgar *et al.* [208] and related comments [209,210].

Although the work of Gutenkunst *et al.* [207] is a valuable contribution which nicely illustrates the difficulties that plague parameter estimation problems in dynamic models, we believe that:

(i) links between sloppiness and previous works on identifiability (which are not cited) should have been established. Our own biased opinion is that identifiability is probably a better framework to analyse the above-mentioned challenges. To begin with, sloppiness seems to lump together lack of structural and practical identifiability. However, structural problems can be addressed by model reformulation or reduction. Practical identifiability problems can be surmounted by more informative data and, ideally, by OED (see related comments by Apgar *et al.* [208]). Consequently, identifiablity seems to be a more powerful concept in the sense that it also provides us with guidelines on how to improve it;
(ii) as model sloppiness can be reduced by the above strategies, it is not a universal property in systems biology. See also Apgar *et al.* [208] for more on this. To be fair, Gutenkunst *et al.* [207] clearly state that ‘universal’ has a technical meaning from statistical physics (a shared property with a deep underlying cause), so universality in this sense does not imply that all models must necessarily share the property. But from conversations with many colleagues, it seems that the latter incorrect meaning has been often assumed; and
(iii) related with (i), the study by Brown & Sethna [189] concludes that sloppiness is not a result of lack of data. Does this imply that it is only related to lack of structural identifiability? We do not get this impression, because e.g. in the study by Apgar *et al.* [208] and related comments [209,210], the issues discussed seem to be only related to practical identifiability.

In a way, sloppiness has created a somewhat pessimistic view towards parameter estimation in dynamic modelling, similar to the one created by the NFL theorem in optimization as described above. But, in this case, there is no theorem, and there are ways to surmount structural and practical identifiability problems (model reformulaton and/or better experiments can indeed lead to good parameter estimations). We believe that an integrative study of sloppiness and identifiability would be very valuable. We also believe that, in many situations, the lack of informative data is the source of such lack of identifiability, because most biological systems of interest are only partially observed and current measurement technologies often result in large errors. However, advances in such technologies, coupled with new ways of introducing perturbations and the use of OED methods, should lead to identifiable dynamic models [208,211], so we should be optimistic about the calibration of these models.

### 3.7. Perspective from information theory

Information theory was initiated by the work of Shannon [61], who was interested in finding fundamental limits to signal-processing processes in communication and compression of data. The so-called sampling theorem (frequently attributed to Shannon & Nyquist [212]) is one of such fundamental results: a signal's information is preserved if it is uniformly sampled at a rate at least two times faster than its Fourier bandwidth (higher frequency). Or, in other words, a time-varying signal with no frequencies higher than *N* hertz can be perfectly reconstructed by sampling the signal at regular intervals of 1/(2*N*) seconds. Therefore, if we do not have a sampling above this threshold, we cannot recover exactly the original signal.

We find again a theorem that establishes a fundamental constraint on what we can infer from data. Once more, this seems to be another case of a pessimistic view that can be avoided if we exploit other information about the system. Indeed, recent work [213–215] showed how sparsity patterns can be used to perfectly reconstruct signals with sampling rates below the Shannon limit. These works have created the burgeoning new field of compressed (or compressive) sensing, which has already seen the publication of a large number of works, not only regarding the methodology and its extensions but also applications. In the case of biological data, they have been successfully applied in bioinformatics [216]. Very recently, Pan *et al*. [217] presented a very interesting compressive sensing approach for the reverse engineering of biochemical networks, assuming fully observed networks. A key question remains open: can we apply this framework to partially observed networks?

### 3.8. Perspective from machine learning

Machine learning, generally considered a subfield of artificial intelligence, aims to build systems (usually programs running on computers) that can learn from data and act according to requirements. In other words, it is based on data-driven approaches where the systems learn from experience (data). Machine learning methods have been widely used in bioinformatics [218,219] and computational and systems biology [220,221]. Traditionally, the data-driven models used in machine learning have been considered on the other side of the spectrum from mechanistic models. However, recent machine learning advances have made it possible to somehow link both and consider the automatic generation of mechanistic models via data-driven methods. In this line, Kell & Oliver [222] argue that data-driven approaches should be regarded as complementary to the more traditional hypothesis-driven programmes.

During the last decade, several studies have been presented which examine the full automation of reverse engineering, from hypothesis generation to experiments and back, in what has been termed machine science [223]. A prominent example is the robot scientist developed by King *et al.* [224–226] and its applications in functional genomics, illustrating how a machine can discover novel scientific knowledge in a fully automatic manner.

An automated process for reverse engineering of nonlinear dynamical systems was presented by Bongard & Lipson [227], illustrating how the method could be used for automated modelling in systems biology, including the automatic generation of testable hypotheses. More recently, Schmidt & Lipson [228] presented an approach to automatically generate free-form natural laws from experimental data. Despite these success stories, the large-scale and partially observed nature of most biological systems will undoubtedly pose major challenges for the widespread application of these procedures in the laboratory.

## 4. Conclusion: lessons from converging perspectives

Reverse engineering can help us to infer, understand and analyse the mechanisms of biological systems. In this sense, modelling is a systematic way to efficiently encapsulate our current knowledge of these systems. However, the value of models can (and should) go beyond their explanatory value: they can be used to make predictions, and also to suggest new questions and hypotheses that can be tested experimentally. Systems biology will succeed if the practical value of theory is realized [5].

The above perspectives from different areas clearly show overlaps and convergent ideas. For example, the ill-posed nature of many identification problems, as described in inverse problem theory, has obvious parallels in optimization (multi-modality, flatness of cost functions), systems identification (lack of identifiability) or CRNT (non-uniqueness). Similarly, some regularization techniques can be regarded as Bayesian approaches where certain prior distributions are enforced. Other overlaps and synergies are not so obvious (e.g. the role of sparsity in inference) and will require careful study.

Several basic lessons can be extracted from the different perspectives that we have briefly reviewed. The first lesson is that modelling should start with questions associated with the intended use. These questions will also help us to choose the level of description that must be selected [229]. We should focus on making the right questions, even if we can only give approximate answers to them (an exact answer to the wrong question is of little use [230]).

The second lesson is that these reverse engineering problems are extremely challenging, so pessimistic views are understandable (e.g. Brenner [231] thinks that they are not solvable). But, as nicely argued by Noble [232], the history of science contains many incorrect claims to impossibility. In fact, we have seen in previous sections that the existence of several pessimistic theorems has not precluded advances in related areas. Brenner [231] cites an article [233] on inverse problems to justify his skepticism. In that work, Tarantola [233] comments on the difficulties that plague inverse problems in geophysics, concluding that observations should not be used to deduce a particular solution but to falsify possible solutions. In our opinion, even if this holds for inverse problems in systems biology (which is questionable), it does not mean that we are doomed to fail (Popper considers science as falsification, and Tarantola's view builds on that). Besides, fortunately, optimistic views are also present in the community, and modern statistical methods are here to help (e.g., see the excellent preface in the book by Stumpf *et al.* [185]).

In this context, it is also worth mentioning that, as described by Silver [41], modelling and simulation have been very successful in some areas (notably, short-term weather forecasts), but have failed dramatically in others (e.g. earthquake predictions). Many decades of research have been invested in both topics. However, in the case of weather, we have better data and a deeper knowledge of the physico-chemical mechanisms involved. The atmosphere and its boundaries are much easier to explore than the tensions and displacements underground. The optimistic system biologist will rejoice in the description of systems biology as cellular weather forecasting [5]. But we should also bear in mind that it took many years to develop the theoretical and computational methods behind current weather models.

The third lesson is that approximate methods can give us rather good solutions to many of these hard problems. Notably, we have seen how randomized algorithms of several types (e.g. stochastic methods for global optimization, or MCMC sampling methods in Bayesian inference) can produce good results in reasonable computation times. Needless to say, this does not mean that deterministic algorithms should be abandoned (e.g. in global optimization, they are making good progress). Rather, it will be very interesting to see how hybrids between deterministic and stochastic methods result in techniques that scale up well with problem size.

The fourth lesson is that, although the Bayesian versus frequentism controversy continues [234], Bayesian methods are probably better suited for many of the inference problems in systems biology. Stumpf *et al.* [185] mention the difficulties of classical statistics with an area that is data-rich but also hypotheses-rich. Incidentally, it is interesting to note that Lindley [235] predicted a Bayesian twenty-first century. However, as it has happened in other areas of science, joining forces might be a strategy worth exploring, as recently illustrated by Raue *et al.* [200].

The fifth lesson is that we need to establish links between identifiability, as developed in the systems and control area, and related concepts developed in other fields, such as sloppiness [207]. The Bayesian view will also help in establishing the practical limits for reverse engineering of kinetic models [236].

The sixth lesson is that we need to exploit the structure of dynamic models. In addition to CRNT, Kaltenbach *et al.* [157] also mention the theory of monotone systems [237] as a promising avenue, highlighting the need of further research to be able to apply these theories to biological networks of realistic complexity.

A final seventh lesson is that, although systems biology is a truly interdisciplinary area, we need to coordinate more efforts and exchange more notes. Different communities have developed theories and tools that have major implications for the identification and reverse engineering of biological systems, but in many cases they have been doing so in isolation from each other. There are several notable examples where collaborations have been very successful, such as SBML [238], BioModels Database [239] or the DREAM challenges [94]. As indicated by Kitano [240], international alliances for quantitative modelling in systems biology might be needed. Whole-cell models will require robust and scalable inference and estimation methods. Much reverse engineering work lies ahead.

## Endnote

1 This distinction between the reduced problem of recovering interactions and the general reverse engineering problem is commonly made. For example, the challenges designed in the DREAM initiative belong to one of these two classes [29]. This entails that constraint-based approaches, which are intermediate between the dynamic and interaction-based models, will not be explicitly considered here. We refer the interested reader to the existing reviews on the subject [22,30–32].
